# Viewpoint: Periprosthetic joint infection and dental antibiotic prophylaxis guidelines

**DOI:** 10.5194/jbji-6-363-2021

**Published:** 2021-10-01

**Authors:** Miao Xian Zhou, Elie F. Berbari, Cory G. Couch, Scott F. Gruwell, Alan B. Carr

**Affiliations:** 1 Department of Dental Specialties, Division of Periodontics, Mayo Clinic, Rochester, MN, USA; 2 Department of Medicine, Division of Infectious Diseases, Mayo Clinic, Rochester, MN, USA; 3 Department of Medicine, Division of Orthopedics, Mayo Clinic, Rochester, MN, USA; 4 Department of Dental Specialties, Division of Prosthodontics, Mayo Clinic, Rochester, MN, USA

## Abstract

The purpose of this viewpoint is to provide a framework that is used within the Mayo Clinic to align recommendations from infectious disease experts, dental specialists, and orthopedic surgeons with regards to need for antibiotic prophylaxis prior to invasive dental procedures.

## 1 Viewpoint

There has been great interest in the use of antibiotic prophylaxis prior to dental procedures to help prevent prosthetic joint infections (PJIs). In the 1970s, preoperative, perioperative, and/or postoperative antibiotics were used sporadically to reduce the risk of infection in patients undergoing orthopedic surgery of long duration in certain situations (Fogelberg et al., 1970). In 1975, Cruess et al. (1975) reported three cases of postoperative hip infection that were likely the result of a distant primary source. The hypothesized causes were an abscessed tooth, a urinary tract infection, and a respiratory tract infection. In 1988, Maderazo et al. (1988) reported a late total joint prosthesis infection rate of 0.6 %, a substantial 7.5-fold increase from a decade earlier at 0.08 % (Maderazo et al., 1988). They found that the three most common origins of infection were the skin and soft tissues (46 %), dental infections (15 %), and urinary tract infections (13 %). They reported that the most common pathogen responsible for late PJIs was staphylococcus (54 %), even when infection was of dental origin. The authors concluded, based on mortality and cost calculations, that chemoprophylaxis was justified for dental procedures.

Subsequently, additional studies have been conducted to examine the relationship between dental procedures and the risk of PJIs. In 1984, a prospective cohort study of 1000 patients receiving 1112 total joint replacements between 1966 and 1980 was published (Ainscow and Denham, 1984). Participants were advised not to take antibiotic prophylaxis before dental or surgical procedures. The result of the study found only three cases (0.27 %) of hematogenous PJI after an average follow-up of 6 years.

In 1987, dental charts of 2693 patients who underwent prosthetic joint surgery were examined and the authors found that of the 30 (1.1 %) late PJIs, there was only one that could be related to dental treatment, comprising 0.04 % of the cases. Additionally, it was noted that gram-positive staphylococci made up 53 % of the isolates (Jacobson and Matthews, 1987).

Other authors found the relationship between dental infections and PJI risk to be higher. In 1997, a retrospective review of records consisting of 3490 patients treated with total knee arthroplasty (TKA) between 1982 and 1993 was performed to examine the risk of infection associated with dental surgery (Waldman et al., 1997). The authors found that 7 out of the 62 TKAs with late infection (6 months after placement) were strongly associated with a dental procedure. These seven cases represent 0.20 % of all TKA procedures performed but comprised 11 % of all infections identified. The authors theorized that patients who have a compromised host defense mechanism (e.g., diabetes and rheumatoid arthritis) and undergo extensive dental procedures (characterized by an average of 115 min; range 75–205 min) may warrant prophylactic antibiotics.

In a widely cited prospective case-control study with 339 case patients and 339 control subjects who underwent TKA or hip arthroplasty during 2001–2006, Berbari et al. (2010) demonstrated that there was no increased risk of PJIs for patients undergoing high- or low-risk dental procedures who were not administered antibiotic prophylaxis (adjusted odds ratio, OR: 0.8; 95 % confidence interval, CI: 0.4–1.6) when compared to the risk for patients not undergoing a dental procedure (adjusted OR: 0.6; 95 % CI: 0.4–1.1) (Berbari et al., 2010). They concluded that the risk of PJI was not increased following dental procedures in patients with hip or knee replacement and is unaffected by antibiotic prophylaxis. This study was rated as the highest-quality study using the Newcastle–Ottawa quality assessment scale of case-control and cohort studies on the topic of PJIs and dental infections (Slullitel et al., 2020). Berbari et al. remarked that reported PJIs due to dental procedures were more likely due to routine daily activities (such as tooth brushing, flossing, or chewing) than by bacteriemia related to dental procedures. The authors agreed with the 2003 statement from the American Dental Association (ADA) along with the American Academy of Orthopaedic Surgeons (AAOS) on the emphasis on maintenance of good oral hygiene to decrease bacteremia from routine daily activities.

## 2 Changes in antibiotic prophylaxis recommendations over time

In light of the growing body of evidence regarding the role of dental procedures on the risk of PJI, the ADA and AAOS have updated their guidance on antibiotic prophylaxis over the years, which has been well-summarized by Goff et al. (2020). The 2012 AAOS/ADA guidelines by Rethman et al. (2013) reached a consensus by the work group that patients with prosthetic joint implants should maintain appropriate oral hygiene due to evidence of the relationship of oral microflora to bacteremia and that this bacteremia may in turn be associated with poor oral hygiene (Rethman et al., 2013). Additionally, the 2016 appropriate use criteria tool constructed by an expert writing panel can also help to guide clinicians on whether antibiotic prophylaxis may be indicated (Quinn et al., 2017). A recent systematic review by Slullitel et al. (2020) revealed no direct evidence to suggest prophylactic antibiotics prior to dental procedures in patients with TJA except in certain situations (Slullitel et al., 2020).

**Table 1:** Mayo Clinic guidelines on prophylactic antibiotics prior to invasive procedures in patients with prosthetic joints.

Prophylactic antibiotics prior to invasive procedures in patients with prosthetic joints		
Mayo Clinic recommendations		
Background		
Given the morbidity and mortality associated with prosthetic joint infection (PJI), patients and providers alike have been eager to do anything possible to prevent this complication. To this end, the use of routine preprocedural (dental, GI endoscopic, and urologic) antibiotic prophylaxis has been widespread. However, there is no evidence to date that the *routine* use of preprocedural antibiotics is an effective method to prevent PJI. Furthermore, any potential benefit of antibiotic prophylaxis must be weighed against the risks of antibiotic toxicity, allergy, and antimicrobial resistance. The following recommendations attempt to balance the risks of PJI with the risks of routine preprocedural antibiotic prophylaxis. Health care providers must always utilize clinical judgement when determining the manner in which these recommendations are applied to their patients. Where possible, patients already taking suppressive antibiotics should be given a different class of medications if preprocedural prophylaxis is deemed necessary.		
Dental procedures (modified AAOS – ADA guidelines 2012)		
It is recommended that patients maintain good oral hygiene, including routine dental care, both before and after joint replacement surgery. The timing of routine dental cleaning should proceed as directed by the dental care provider in order to maintain good oral hygiene. **When possible, it is recommended to avoid elective dental procedures, such as orthognathic surgery, orthodontics, or implant placement for the first 6 months following surgery. However, patients with symptomatic dental conditions should seek immediate evaluation and treatment.** **Antibiotic prophylaxis prior to dental procedures is recommended for all patients within 1 year of surgery.** Antibiotic prophylaxis is not recommended beyond 1 year of surgery, except for select patients, in whom either the risk of hematogenous seeding may be elevated, and/or the consequences of PJI are especially devastating. – Surgical factors: • Prior PJI • Complex Joint Reconstruction (e.g., tumor prostheses) – Medical factors: • Immunosuppression • Hematologic malignancy (e.g., leukemia, lymphoma) • Chemotherapy • DMARDs Antibiotic options for antibiotic prophylaxis prior to invasive dental procedure to be administered 1 h prior to the start of the procedure:		
No drug allergies	Allergic to penicillins or cephalosporins	Unable to tolerate oral medications
Cephalexin or amoxicillin (2 g)	Clindamycin (600 mg orally) Azithromycin or clarithromycin (500 mg orally)	Cefazolin (1 g IV or IM) Clindamycin (600 mg IV)

## 3 PJI guidelines at the Mayo Clinic with recommendations and take-home points

At the Mayo Clinic, the Department of Orthopedic Surgery routinely cares for medically complex and compromised patients. In 2015, a set of guidelines was developed in collaboration with the departments of Dental Specialties, Orthopedic Surgery, and Infectious Diseases to help provide practical and judicious use of antibiotics to patients undergoing dental procedures, particularly in patients with significant comorbidities. In these recommendations (Table 1), it was advised to use dental antibiotic prophylaxis for 1 year following joint arthroplasty (including maintenance or prophylaxis). The guidance also recommended a deliberate hold on all elective dental procedures for the first 6 months after joint surgery. The rationale for the one-year mark is due to an internal study conducted at the authors' institution that showed that the risk of hematogenous seeding is higher in the first year after total joint arthroplasty.

After the first year, dental antibiotics prophylaxis is not recommended for most patients. Patients belonging to high-risk categories, such as those individuals who are immunosuppressed (e.g., hematologic malignancy such as leukemia, lymphoma), those undergoing chemotherapy, those utilizing disease-modifying medications for rheumatoid arthritis, or in cases of prior PJIs or complex joint reconstruction (e.g., tumor prosthesis), antibiotic prophylaxis beyond 1 year may be recommended. If a patient is already taking antibiotics for chronic suppression, the provider may wish to consider a macrolide or clindamycin as an alternative option for antibiotic prophylaxis if deemed necessary, with mindfulness of the increased risk for *Clostridium difficile* such as with the use of clindamycin. However, if the patient's antibiotic regimen covers common dental flora (i.e., amoxicillin or Keflex), then the patient may be able to time their routine suppressive dose just an hour prior to the anticipated invasive dental procedure to adhere to antibiotic stewardship. Consideration for additional antibiotic therapy is warranted if their chronic suppressive antibiotic is not one that is recommended for dental prophylaxis. Procedures may be postponed or canceled if signs of oral infections are present, and dental consultations are sought on a case-by-case basis.

## 4 Conclusion

It is important to note that current studies demonstrate no association of PJI following invasive dental procedures, and many of the studies exploring this topic are of low quality. Antibiotic stewardship is necessary to assist in combatting the rise of antibiotic resistance through patient education and individualized risk assessment on patients undergoing dental procedures that have had prosthetic joint surgery. Clear and concise communication regarding antibiotic recommendations and risk of PJIs prior to, during, and after joint surgery is essential. Optimum oral health plays an important role in the reduction of the risk. Emphasis should be placed on maintaining optimal oral health to reduce the risk of infection and to minimize the patient's risk of PJI, especially in patients with significant comorbidities.

## Data Availability

No data sets were used in this article.
